# Adherence to Mediterranean Diet and Tendency to Orthorexia Nervosa in Professional Athletes

**DOI:** 10.3390/nu14020237

**Published:** 2022-01-06

**Authors:** Dinko Martinovic, Daria Tokic, Lovre Martinovic, Marino Vilovic, Josip Vrdoljak, Marko Kumric, Josipa Bukic, Tina Ticinovic Kurir, Marino Tavra, Josko Bozic

**Affiliations:** 1 Department of Pathophysiology, University of Split School of Medicine, 21000 Split, Croatia; dinko.martinovic@mefst.hr (D.M.); lm71805@mefst.hr (L.M.); marino.vilovic@mefst.hr (M.V.); josip.vrdoljak@mefst.hr (J.V.); marko.kumric@mefst.hr (M.K.); tticinov@mefst.hr (T.T.K.); 2 Department of Anesthesiology and Intensive Care, University Hospital of Split, 21000 Split, Croatia; dtokic@kbsplit.hr; 3 Department of Pharmacy, University of Split School of Medicine, 21000 Split, Croatia; josipa.bukic@mefst.hr; 4 Faculty of Kinesiology, University of Split, 21000 Split, Croatia; martav@kifst.hr

**Keywords:** professional athletes, orthorexia nervosa, Mediterranean diet, physical activity, IPAQ, MDSS, ORTO-15

## Abstract

Among many lifestyle components that professional athletes have to follow, nutrition is gradually growing to be one of the key factors for achieving and maintaining optimal sport performance. The Mediterranean diet (MD) is recognized as one of the healthiest dietary patterns worldwide; however, data regarding adherence to the MD among professional athletes are still scarce. Moreover, with the imposed need for a healthy diet among professional athletes, orthorexia nervosa (ON) could become a rising issue. This cross-sectional study included 150 professional athletes and 150 matched recreational athletes from Croatia. Four questionnaires were used for the assessment: general information, a test for the diagnosis of ON (ORTO-15), the International Physical Activity Questionnaire (IPAQ) and the Mediterranean Diet Serving Score (MDSS). Significantly more professional athletes were adherent to the MD (*p* < 0.001) and had a tendency to ON (*p* < 0.001). Moreover, there was a significant negative correlation between the ORTO-15 score and the total MET min/week score (r = −0.524, *p* < 0.001) and a significant positive correlation between the MDSS score and the total MET min/week score in the professional athlete group (r = 0.478, *p* < 0.001). All of these results imply that professional athletes are more concentrated on their dietary patterns than recreational athletes, and that due to this dedication, they possibly have a higher adherence to the MD but also possibly a higher risk for developing ON. However, the association between ON and the MD should be further addressed in the future.

## 1. Introduction

In recent decades, the rapid development of science and our knowledge regarding human physiology has had a major impact on the lifestyle of professional athletes. Among many lifestyle components that professional athletes have to follow, nutrition is gradually growing to be one of the key factors for achieving and maintaining optimal sport performance [1,2,3]. In addition to planning everyday meals, special focus is also placed on the nutritional needs of athletes before, during and after their exercise [4]. However, due to a wide range of different sports and individual needs, it is impossible to determine a perfect dietary pattern that all professional athletes should follow. Nevertheless, some dietary patterns have emerged as more beneficial than others [5].

Even though many diets today are created by nutritionists, some of them are cultural and based on traditional cuisine. The Mediterranean diet (MD) is the traditional cuisine of countries on the shores of the Mediterranean Sea, and it is recognized as one of the healthiest dietary patterns worldwide [6,7]. The MD consists of a high intake of olive oil, fruits, vegetables, nuts, cereals and legumes, less frequent intake of fish, eggs and dairy products and occasional intake of sweets and meat, with predominantly white meat over red meat [8]. Red wine is recommended every day in a small amount which depends on the sex [9]. The MD is globally one of the most studied dietary patterns due to its major health benefits [10]. Studies have shown that higher adherence to the MD is associated with a reduced risk of developing cardiovascular diseases, diabetes, obesity, cognitive diseases and depression [11,12,13,14,15]. Recent findings showed evidence that the MD has a lipid-lowering effect, protects against oxidative stress, inflammation and platelet aggregation, regulates signals involved in the pathogenesis of cancer, inhibits nutrient sensing pathways by specific amino acid restriction and influences metabolic health through gut microbiota [16,17].

However, with the rise in awareness about the benefits of healthy nutrition, a new issue has emerged. Orthorexia nervosa (ON) was first defined more than 20 years ago as a pathological eating condition where people have a compulsive obsession with healthy food choices [18]. They follow radical diet habits due to which they may develop issues in everyday social life, nutritional deficiencies, severe weight loss and other health problems associated with eliminating certain foods [19]. ON has become a major global issue in the last two decades with the rise in awareness about the benefits of healthy nutrition [20]. Moreover, the infliction of the perfect body image through media and the internet has further boosted ON among the younger population [21]. Nevertheless, ON is not classified as an eating disorder due to still undetermined symptoms and risk factors, and, subsequently, its uncertain diagnosis.

Data regarding adherence to the MD and the tendency to ON among professional athletes are still very scarce and inconclusive. Several studies that investigated the MD among professional athletes found contradictory results as some of them showed high adherence, while others found very low adherence to the MD [22,23,24,25,26]. However, these studies were very heterogeneous as they were conducted in different regions and on different sport groups, and they used different instruments to measure adherence. Moreover, a question was raised concerning whether the MD provides the appropriate nutrients to professional athletes since it minimizes meat consumption, with subsequent lower protein intake [27]. However, even though this thesis is somewhat correct, the MD still provides other beneficial nutrients, and since it is considered one of the healthiest diets, it is interesting to find out whether athletes adhere to it. On the other hand, with the imposed need for a healthy diet among professional athletes, ON could become a rising issue. Several studies which investigated the ON tendency in professional athletes implied that they could have a high risk of developing ON [28,29,30].

Therefore, the aim of this study was to evaluate adherence to the MD and the tendency to ON among professional athletes in Croatia. Moreover, a secondary goal was to compare these results with matched recreational athletes and to explore the possible association between adherence to the MD, tendency to ON and the level of physical activity.

## 2. Materials and Methods

### 2.1. Study Design

This cross-sectional survey-based study was performed at several fitness centers in Croatia during the time period from September 2021 to November 2021.

The study was conducted in accordance with the latest Helsinki Declaration, and it was approved by the Ethics Committee of the University of Split School of Medicine (No: 003-08/20-03/0005, Date: 3 July 2020). All subjects consented to participate by submitting the completed questionnaire.

### 2.2. Subjects

The survey was conducted on 150 professional athletes and 150 recreational controls. Participants were recruited through fitness coaches and acquaintances. The survey was performed using the online program Google Forms^®^ which was distributed using QR codes and email. 

The inclusion criteria for professional athletes were: 18–40 years of age and active involvement in professional sports. Professional sport involvement was defined as being registered as a professional sportsperson for a certain sport, competing in professional competitions and training for at least 5 times a week. The exclusion criteria were: a pause in professional sports longer than 3 months and involvement in any predominantly non-physical sport. The participants we included were involved in judo, boxing, kickboxing, soccer, water polo, power lifting, handball, athletics, rugby, basketball, tennis, volleyball and dancing. 

The inclusion criteria for recreational athletes were: 18–40 years of age and >2 times per week active involvement in recreational sports. The exclusion criteria were: involvement in any professional sport and recreational involvement in any predominantly non-physical sport.

### 2.3. Questionnaires

The survey consisted of four components. The first part was the general information questionnaire which explored basic data about the participants such as age, sex, involvement in professional sport and anthropometric characteristics. This questionnaire was developed using the most relevant literature for the purpose of this study, and it included 12 questions.

The second part of the survey was the International Physical Activity Questionnaire—Short Form (IPAQ-SF). This questionnaire is a reliable open-ended tool used for evaluating physical activity, and it was validated in the Croatian language [31,32]. The IPAQ-SF assesses self-reported physical activity of four intensity levels: vigorous-intensity activities; moderate-intensity activities; walking; and sitting. The IPAQ-SF’s authors suggested that observational studies should use the “last 7 days recall” version. The results of the IPAQ-SF were used to calculate MET (metabolic equivalent of task) minutes per week (min/week) scores according to the following formulas [33]:

- Walking MET min/week = 3.3 × walking minutes × walking days
- Moderate MET min/week = 4.0 × moderate activity minutes × moderate days
- Vigorous MET min/week = 8.0 × vigorous activity minutes × vigorous days
- Total MET min/week = walking + moderate + vigorous MET min/week scores

The third part of the survey was the Mediterranean Diet Serving Score (MDSS). The MDSS is a reliable 14-item questionnaire which was validated in the Croatian language [34,35]. The MDSS is used to assess adherence to the MD, and it is updated by the latest guidelines of the Mediterranean Diet Pyramid, based on the frequency of consuming certain foods and food groups and scoring them by one (1), two (2) or three (3) points, depending on the recommendation of intake. Three-point foods are fruits, vegetables, cereals and olive oil, which are recommended to be consumed every meal. Two-point foods are nuts, which are recommended to be consumed daily, and dairy products, which are recommended to be consumed every meal. Lastly, one-point foods are white meat, red meat, fish, potatoes, legumes, eggs, sweets and wine, which should be consumed several times a week, the exact number depending on the certain food group. The cutoff value for determining adherence to the MD is a total MDSS score of ≥14 points.

The last part of the survey was the ORTO-15 questionnaire. The ORTO-15 questionnaire is a reliable and validated 15-item questionnaire used for assessing pathological obsession with healthy food [36]. It is self-reported, and all questions are scored on a four-point Likert scale in a range from “always” to “never”. The total score range is 15–60 points, and a lower score is suggestive of a higher pathology. The cutoff value of <40 is indicative of ON, as suggested by the questionnaire’s authors [34]. However, several more recent studies recommended that the abovementioned cutoff leads to an exaggerated prevalence of false positive scores [37,38,39]. Hence, they proposed the cutoff value of <35, which they believe is more specific and reliable. Accordingly, the cutoff value for ON in this study was set at <35 points in ORTO-15.

### 2.4. Survey Pre-Testing

The ORTO-15 questionnaire was assessed and back translated to the Croatian language by an expert who has a degree in both English and Croatian languages. Pre-testing of the whole survey was conducted on 20 randomly chosen professional athletes and 20 randomly chosen recreational athletes. The average time needed to complete the survey was 12–15 min. The feedback from the responders determined that all the questions were clear and understandable. The internal consistency of the ORTO-15 in this pre-testing was acceptable as Cronbach’s alpha coefficient was 0.80.

### 2.5. Statistical Analyses

Analyses of the data was performed using the computer software MedCalc (MedCalc Software, Ostend, Belgium, version 17.4.1). Qualitative variables were presented as whole numbers and percentages, while quantitative data were presented as mean ± standard deviation or median and interquartile range. The normality of the data distribution was estimated using the Kolmogorov–Smirnov test. Qualitative data were compared between groups using the chi-square test. Parametric data were compared between groups using the Student t-test, while the non-parametric data were compared using the Mann–Whitney U test. Correlation analyses were performed using the non-parametric Spearman correlation method. Comparison between correlation coefficients was performed with Fisher’s z transformation. The level of statistical significance was set at a *p*-value of <0.05.

## 3. Results

This study included 165 (55.0%) male participants and 135 (45%) female participants. The mean age of the study population was 24.2 ± 4.8 years. The professional athletes had a significantly higher body weight (84.1 ± 16.5 vs. 77.6 ± 13.8 kg, *p* < 0.001), body height (183.2 ± 10.8 vs. 177.4 ± 9.2 cm, *p* < 0.001) and BMI (24.8 ± 3.3 vs. 23.8 ± 3.0 kg/m^2^, *p* < 0.001) compared to the recreational athletes. Moreover, there was a statistically significant difference regarding the education level as most professional athletes finished high school (36.7%), while most recreational athletes had a master’s degree (50.7%). Most of the professional athletes were engaged in fighting sports (44.7%) (Table 1).

**Figure 1:**
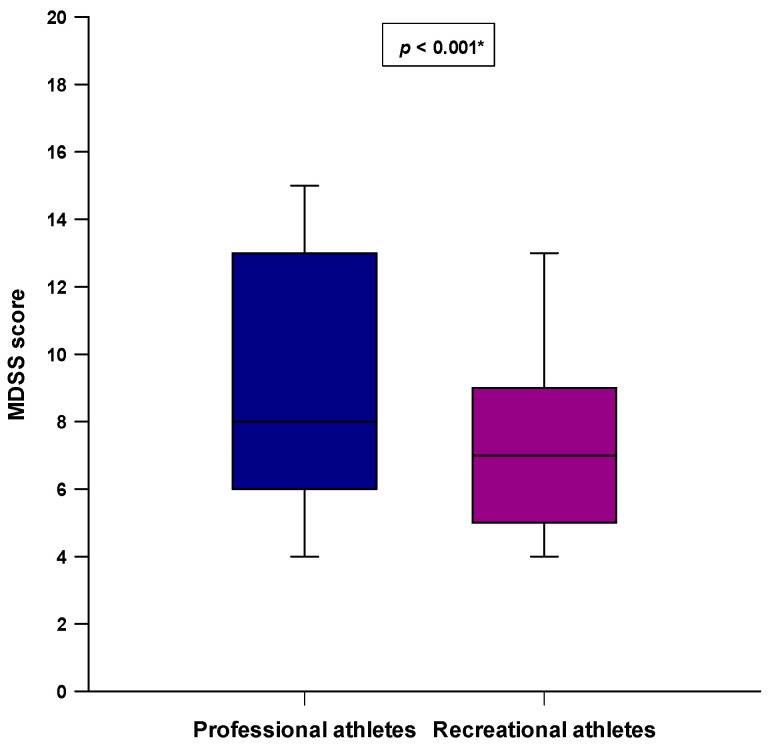
Difference in the total MDSS score between the professional athletes (*N* = 150) and recreational athletes (*N* = 150). * Mann–Whitney U test.

**Figure 2:**
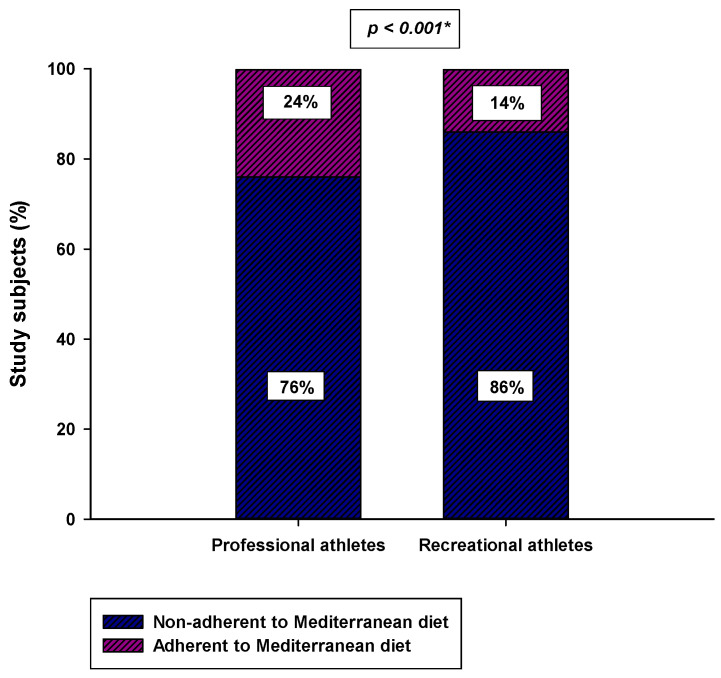
Difference in the number of participants adherent to the MD (MDSS score ≥ 14) between the professional athlete group (*N* = 150) and the recreational group (*N* = 150). * Chi-square test.

Professional athletes had a significantly higher total MDSS score compared to the recreational athletes (8.0 (6.0–13.0) vs. 7.0 (5.0–9.0); *p* < 0.001) (Figure 1). Moreover, a significantly higher number of professional athletes were adherent to the MD (MDSS ≥ 14) compared to the recreational athletes (36 (24%) vs. 21 (14%); *p* = 0.039) (Figure 2).

Regarding the specific food components of the MDSS, professional athletes had significantly higher adherence to olive oil (*p* < 0.001), vegetables (*p* < 0.001), dairy (*p* = 0.014), fish (*p* = 0.017), red meat (*p* < 0.001) and sweets (*p* < 0.001) compared to the recreational athletes (Table 2). On the other hand, recreational athletes had significantly higher adherence to legumes (*p* < 0.001), white meat (*p* < 0.001) and wine (*p* < 0.001) compared to the professional athletes (Table 2).

**Figure 3:**
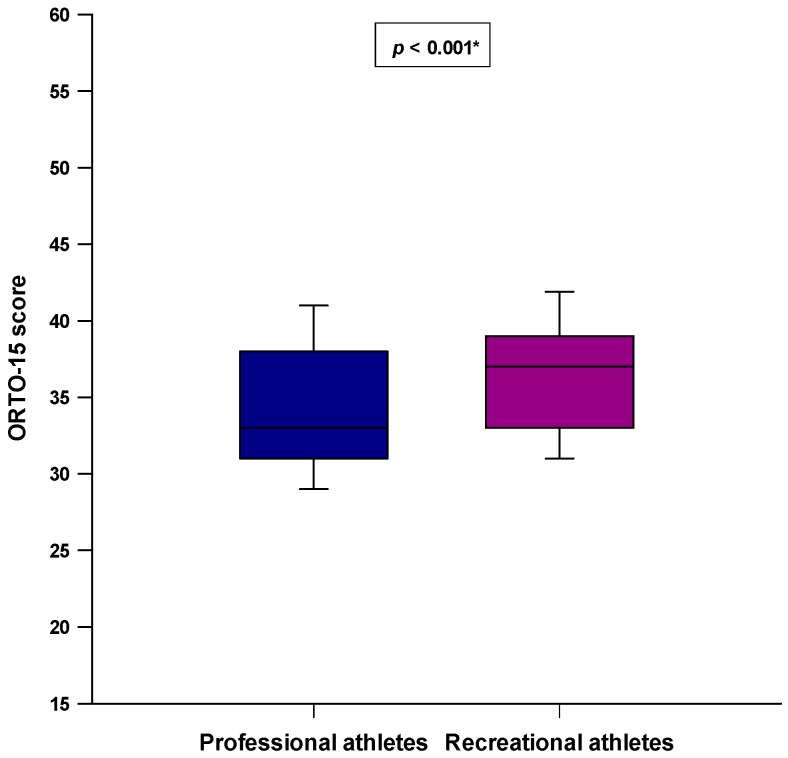
Difference in the ORTO-15 score between the professional athlete (*N* = 150) and recreational athlete groups (*N* = 150). * Mann–Whitney U test.

**Figure 4:**
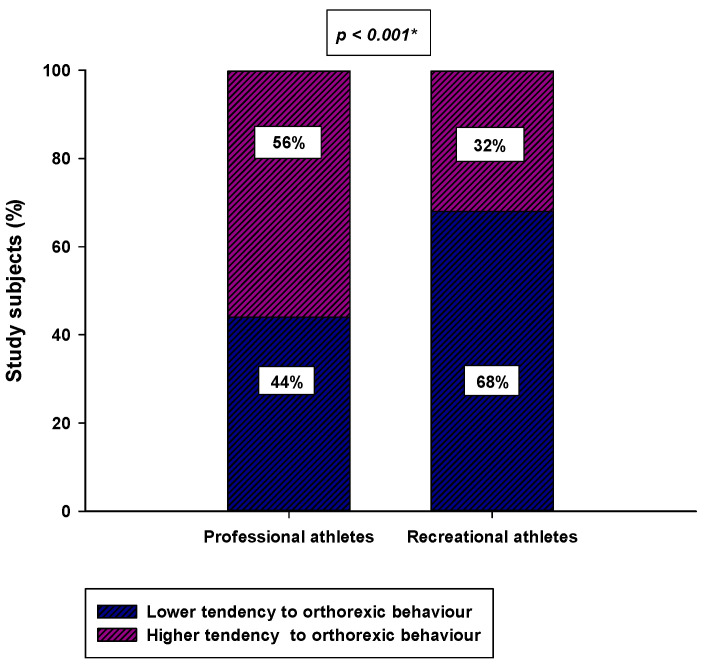
Difference in the number of participants with a tendency to ON (ORTO-15 score <35) between the professional athlete group (*N* = 150) and the recreational group (*N* = 150). * Chi-square test.

Professional athletes had a significantly lower ORTO-15 score compared to the recreational athletes (33.0 (31.0–38.0) vs. 37.0 (33.0–39.0); *p* < 0.001) (Figure 3). Furthermore, a significantly higher number of professional athletes had a tendency to ON (ORTO-15 < 35) compared to the recreational athletes (84 (56%) vs. 48 (32%); *p* < 0.001) (Figure 4).

**Figure 5:**
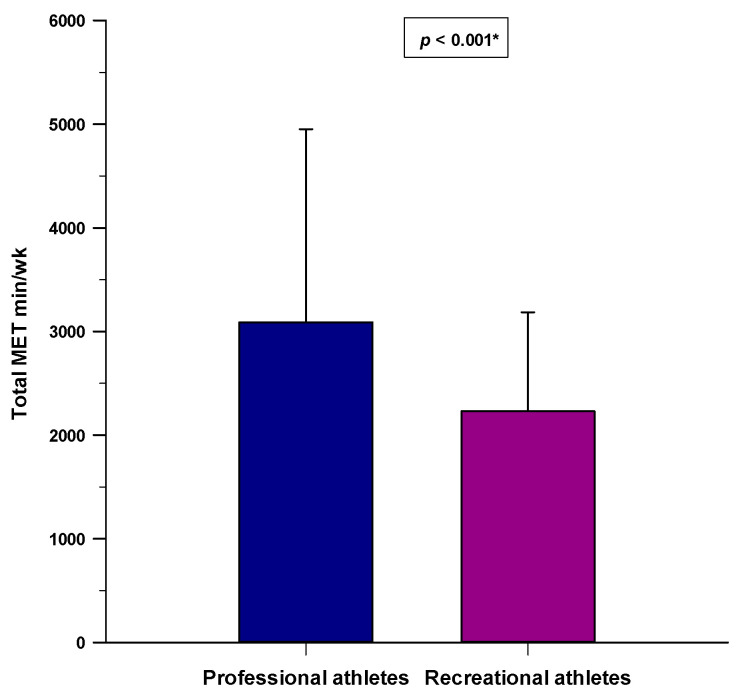
Difference in the total MET min/week score between the professional athlete (*N* = 150) and recreational athlete groups (*N* = 150). * Student’s *t*-test.

Professional athletes had a significantly higher total MET min/week score compared to the recreational athletes (3089.7 ± 1861.9 vs. 2228.6 ± 957.1 MET min/week; *p* < 0.001) (Figure 5). Moreover, professional athletes had a higher MET min/week score regarding vigorous (*p* < 0.001)- and moderate (*p* < 0.001)-intensity activities compared to the recreational athletes, while there were no differences regarding walking-intensity activities (*p* = 0.455).

**Figure 6:**
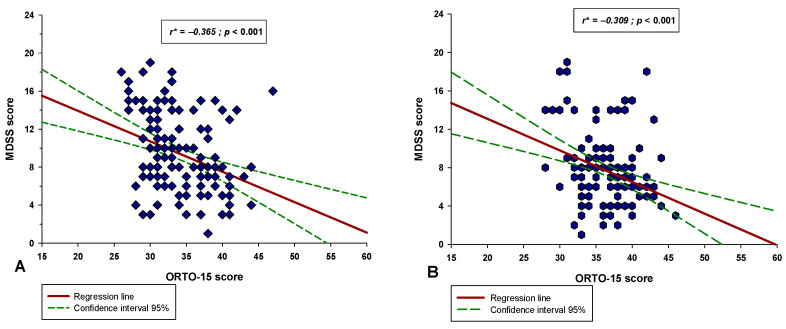
Correlation between the MDSS score and the ORTO-15 score in the (**A**) professional athlete group (*N* = 150) and in the (**B**) recreational athlete group (*N* = 150). * Spearman’s correlation coefficient.

**Figure 7:**
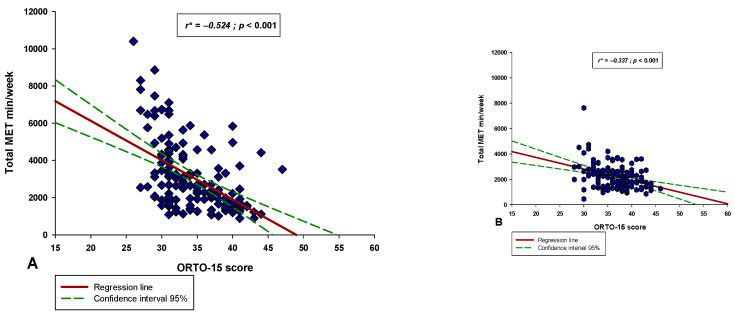
Correlation between the total MET min/week score and the ORTO-15 score in the (**A**) professional athlete group (*N* = 150) and in the (**B**) recreational athlete group (*N* = 150). * Spearman’s correlation coefficient.

**Figure 8:**
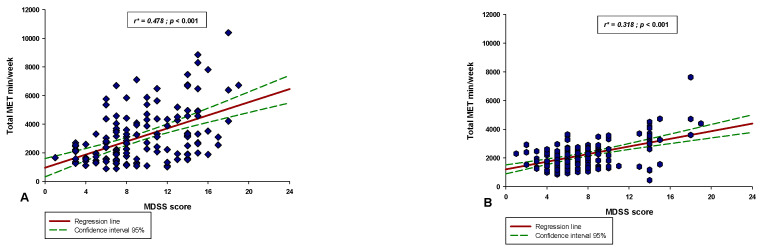
Correlation between the total MET min/week score and the MDSS score in the (**A**) professional athlete group (*N* = 150) and in the (**B**) recreational athlete group (*N* = 150). * Spearman’s correlation coefficient.

There was a significant negative correlation between the MDSS score and the ORTO-15 score in both the professional athlete group (r = −0.365, *p* < 0.001) and the recreational group (r = −0.309, *p* < 0.001) (Figure 6). Additionally, these two correlations were similar, and the coefficients did not significantly differ between them (test statistic z = 0.542; *p* = 0.294). Moreover, there was a significant negative correlation between the ORTO-15 score and the total MET min/week score in both the professional athlete group (r = −0.524, *p* < 0.001) and the recreational group (r = −0.337, *p* < 0.001) (Figure 7). Lastly, there was a significant positive correlation between the MDSS score and the total MET min/week score in both the professional athlete group (r = 0.478, *p* < 0.001) and the recreational group (r = 0.318, *p* < 0.001) (Figure 8).

Furthermore, multivariable logistic regression showed that the total MET min/week score (OR 1.0005, 95% CI 1.0003–1.0008, *p* < 0.001) and the MDSS score (OR 1.0836, 95% CI 1.0059–1.1672, *p* = 0.034) were significant predictors of high tendencies to ON behavior when computed along with baseline characteristics (Table 3).

## 4. Discussion

This study shows that 24% of professional athletes are adherent to the MD in our sample and that professional athletes had a statistically higher total MDSS score compared to the control recreational athletes. Moreover, 56% of the professional athletes had a tendency to ON behavior, and they had a statistically lower ORTO-15 score compared to the control recreational athletes. Additionally, we found a significant negative correlation between the ORTO-15 score and the MDSS score. However, it is important to note that a lower ORTO-15 score represents a greater tendency towards ON.

As mentioned previously, the MD is known for its benefits on health, which could explain why athletes, both professional and recreational, would strive towards this diet regime [40]. Our study shows that professional athletes had significantly higher adherence to this diet pattern than recreational athletes. Our study is in line with other similar studies conducted on this specific group. A study by Alacid et al. investigated adherence to the MD in 90 young female kayakers in Spain, where they found medium and excellent adherence to the MD in 42.2% and 56.6% of the participants, respectively [22]. Another study conducted on amateur endurance cyclists showed higher adherence to the MD in comparison to the control group [25]. On the other hand, some studies found contradictory findings when it comes to professional athletes and the MD. A study by Sanchez-Benito et al. found low to no adherence to the MD in young cyclists [23]. Another Spanish study conducted on elite female futsal athletes also showed lower adherence to the MD [24]. One of the possible explanations is lack of education and abandonment of the traditional MD, leaning towards influences of other cultures and lifestyles. Choosing proper nutrients is as important as regular exercise, especially when it comes to professional sports. To be an elite athlete demands certain sacrifices in order to achieve the best results. As their career in professional sports depends on their performance, it is easy to predict that they will nurture their bodies with the best possible nutrients in order to maintain their health and be prepared for all the challenges in their career. 

Furthermore, our study found that professional athletes were more adherent to olive oil, vegetables, fish, red meat and sweets, while recreational athletes were more adherent to white meat, wine and legumes. These results could be explained through the different requirements of these groups. Professional athletes are in need of “good” fats, higher protein intake and low to no processed sugar. Interestingly, high adherence was found for olive oil and fish, while low adherence was found for white meat, which may implicate that white meat is their lead source of protein. The low adherence to wine, although known for its antioxidant properties, can be explained by the general avoidance of alcohol in professional athletes. On the other hand, recreational athletes are not that strict in their diet. The high adherence to white meat can be explained by using mostly red meat as their protein source. Furthermore, the low adherence to sweets also suggests their freer dietary style. It is also important to note that recreational athletes had a higher level of education when compared to the professional athlete group. One of the possible explanations is that professional sport requires a lot more dedication, sacrifice and focus in order to compete at a high level. Moreover, as the professional group had higher adherence to the MD, it is possible to assume that level of education is not necessarily crucial when it comes to knowledge about a proper and healthy diet.

Another important finding in our study is the significant positive correlation between physical activity and the MD. In other words, all our participants with a higher total MET min/week score are more adherent to the MD, which is also in line with other studies that found a significant association between physical activity and adherence to the MD [6,41,42]. Our previous Croatia-based study conducted on recreational fitness center users showed a significant association between physical activity and adherence to the MD [6]. Moreover, after dividing our sample into tertiles based on the IPAQ-SF score, it was determined that the third tertile (MET > 3150 min/week) had the most fitness center users (34.4%) which were adherent to the MD, while the first tertile (MET < 1750 min/week) had the least (6.1%). All of the abovementioned findings imply that the MD is important as a dietary style, and it is often chosen as an eating pattern in order to improve both health and sport results. However, the implied association between physical activity and adherence to the MD should be further explored. 

Previous studies conducted on professional athletes showed that they have a higher tendency for ON behavior. A recent Polish study performed on elite athletes using the ORTO-15 questionnaire and the <35 cutoff value showed that 40% of them had a high tendency towards ON behavior [29]. Moreover, an Italian study conducted on professional athletes in endurance sports showed a high percentage of ON tendencies in the sample, and it found that the level of physical activity could be associated with ON behavior [28]. These results are in alignment with our outcome that the ORTO-15 score is in a significant negative correlation with the total MET min/week score. This implies that a higher level of physical activity could possibly be a risk factor for developing ON. Recently, a study by Clifford et al. compared students involved in university sport teams and non-athletic students [43]. They did not find a significant difference between the two groups; however, they did determine that a higher volume of exercise significantly affects the risk for ON behavior. Moreover, recently, a study was conducted in Turkey on patients with obsessive compulsive disorder (OCD), physically active healthy controls and non-active controls [44]. The findings showed that physically active individuals had the highest ON tendencies between the three groups. All of the aforementioned outcomes suggest that there is a strong link between the level of physical activity and ON tendencies. It is sufficiently established that engagement in physical activity is associated with a higher awareness of the importance of nutrition. Likewise, it is possible that the same awareness could possibly develop tendencies to ON behavior. However, ON is still not considered a psychiatric diagnosis, as opposed to the other “traditional” eating disorders, and unlike them, it is still unclear what are the symptoms and risk factors for developing this disorder. 

Furthermore, even though we did not find any significant differences between sexes, in multivariable logistic regression, the female sex may have had a larger predictive value for ON behavior, but due to the large confidence interval (CI), this effect was non-significant. The large CI is due to the pronounced difference in ON tendencies between females engaged in professional sport and those that are not. It is important to note that previous studies demonstrated that ON tendencies are similar in male and female athletes, whereas in the general population, women tend to have higher ON tendencies [29,43]. This is because in making food choices, athletes place factors influencing their performance (nutrient composition, for instance) above the factors relating to the sensory value of food, and most athletes are aware that optimal nutrition is an essential and integral element of a training program. Nevertheless, future studies are needed to deepen this subject.

Lastly, there was a significant positive correlation between the tendency to ON and the MD. As the main characteristic of ON is an overfocus on the “purity” of food to achieve and maintain health, it is possible that people with ON will be more adherent to an already well-established healthy dietary pattern. As previously mentioned, the MD is known for its benefits on health, such as prevention of developing several diseases; therefore, it is possible to assume that adherence to the MD would be higher in people with a tendency to ON behavior. Furthermore, as correlations between ON and the MD were similar in the professional and recreational athlete groups, we can assume that in the current scenario, both groups share some similar psychological traits, and both recognized the MD diet as a possible foundation of a healthy diet. A study conducted by Strahler et al. investigated ON in a survey-based study conducted on mostly younger participants [19]. Only 3.8% of the subjects showed significant signs of orthorexic eating, and they also showed high adherence to the MD. However, again, the limitation of this study was the incomparability of the studied groups, where the non-ON group was composed of 96.2% of the included subjects. To the best of our knowledge, there are no other studies which investigated the connection between ON and adherence to the MD, which certainly is an interesting and important topic.

There are several limitations to our study. Firstly, its cross-sectional design prevents any causal conclusions. Moreover, we were not able to eliminate all the confounding effects which could have interfered with the results. There was a significant difference in the education level of the included participants, which may have possibly influenced the results. Additionally, the study included a heterogeneous group of professional athletes which could possibly interfere with more clear significances in the results. Furthermore, the study was based on several self-reported questionnaires, meaning it is possible that some of the participants provided dishonest or biased answers. Moreover, since the IPAQ-SF is centered on the “last 7 days recall” method, and the MDSS is based on recalling dietary patterns, it is possible that some of the subjects failed to recollect the correct information. However, we believe that due to the sport culture and high ethics among athletes, all the participants provided honest and correct answers in the survey. Lastly, since the ORTO-15 showed an unstable factorial structure, and it was recommended not to use it for evaluating the prevalence of ON, we decided to use it just for grouping our sample into higher and lower tendencies to ON behavior. Moreover, the ORTO-15 has never been validated for evaluation among professional athletes, and recently, it was suggested that it is not acceptable for comparison of different populations/groups. However, since the primary goal of our study was the comparison of professional and recreational athletes in the same population, and we avoided the use of a cutoff for the prevalence of ON, we believe that these limitations were reduced.

## 5. Conclusions

In conclusion, our results show that almost every fourth professional athlete in our study sample is adherent to the MD, and that professional athletes have significantly higher adherence to the MD compared to recreational athletes. Additionally, professional athletes had significantly higher tendencies to ON behavior compared to the recreational athletes. Lastly, we found a significant association between the level of physical activity with both adherence to the MD and tendencies to ON behavior, but this effect was similarly pronounced in both professional and recreational athletes. Hence, all of these results imply that professional athletes are more concentrated on their dietary patterns than recreational athletes, and that due to this dedication, they possibly have a higher risk for developing ON. These outcomes suggest that professional athletes should be further educated about nutrition and dietary plans, but they should also have a psychological support to overcome the pressure of achievement that burdens them.

## Figures and Tables

**Table 1:** Baseline characteristics of the study sample.

Parameter	Study Sample *N* = 300	Professional Group *N* = 150	Recreational Group *N* = 150	p *
Male sex (*N*, %)	165 (55.0)	87 (58.0)	78 (52.0)	0.353
Age (years)	24.2 ± 4.8	24.5 ± 4.0	24.0 ± 5.5	0.314
Weight (kg)	80.8 ± 15.5	84.1 ± 16.5	77.6 ± 13.8	<0.001
Height (cm)	180.3 ± 10.4	183.2 ± 10.8	177.4 ± 9.2	<0.001
BMI (kg/m^2^)	24.2 ± 4.4	24.8 ± 3.3	23.8 ± 3.0	<0.001
Education level				
Elementary school (*N*, %)	2 (0.7)	2 (1.3%)	0 (0)	<0.001
High school (*N*, %)	108 (36.0)	55 (36.7)	53 (35.3)	
Bachelor’s degree (*N*, %)	73 (24.3)	52 (34.7)	21 (14.0)	
Master’s degree (*N*, %)	117 (39.0)	41 (27.3)	76 (50.7)	

All the data are presented as whole numbers (percentages) or mean ± SD. Abbreviations: BMI—body mass index. * Chi-square test or Student’s *t*-test.

**Table 2:** Adherence to MDSS components and differences between groups.

Parameter	Study Sample *N* = 300	Professional *N* = 150	Recreational *N* = 150	p *
Cereals (*N*, %)	61 (20.3)	27 (18.0)	34 (22.7)	0.389
Potatoes (*N*, %)	272 (90.7)	135 (90.0)	137 (91.3)	0.842
Olive oil (*N*, %)	70 (23.3)	52 (34.7)	21 (14.0)	<0.001
Nuts (*N*, %)	104 (34.7)	56 (37.3)	48 (32.0)	0.395
Fruits (*N*, %)	68 (22.7)	42 (28.0)	28 (18.7)	0.076
Vegetables (*N*, %)	80 (26.7)	52 (34.7)	32 (21.3)	0.014
Dairy (*N*, %)	64 (21.3)	45 (30.0)	19 (12.7)	<0.001
Legumes (*N*, %)	221 (73.7)	95 (63.3)	126 (84.4)	<0.001
Eggs (*N*, %)	171 (57.0)	79 (52.7)	93 (62.0)	0.129
Fish (*N*, %)	182 (60.7)	102 (68.0)	81 (54.0)	0.017
White meat (*N*, %)	249 (83.0)	110 (73.3)	142 (94.7)	<0.001
Red meat (*N*, %)	76 (38.0)	57 (38.0)	22 (14.7)	<0.001
Sweets (*N*, %)	143 (47.7)	95 (63.3)	52 (34.7)	<0.001
Wine (*N*, %)	15 (5.0)	1 (0.7)	15 (10.0)	<0.001

All the data are presented as whole numbers (percentages). * Chi-square test.

**Table 3:** Multivariable logistic regression analysis of independent predictors of high tendencies to ON behavior (ORTO-15 < 35).

Parameter	OR	95% CI	*p*
Age	1.025	0.971 to 1.081	0.369
Female sex ^1^	1.401	0.826 to 2.376	0.210
BMI	0.994	0.938 to 1.053	0.834
MDSS score	1.074	0.997 to 1.058	0.049
Total MET min/week	1.0005	1.0003 to 1.0007	<0.001
Professional athlete group ^2^	1.966	1.160 to 3.332	0.012

^1^ Reference group is male subjects. ^2^ Reference group is recreational athletes. Abbreviations: MDSS—Mediterranean Diet Serving Score; OR—multivariable adjusted odds ratio; 95% CI—95% confidence interval; BMI—body mass index; MET—metabolic equivalent of task.

## Data Availability

The data supporting the reported results are available upon request to the corresponding author.
